# Actin Comets: Traversing the Cellular Universe

**DOI:** 10.1371/journal.pbio.1001766

**Published:** 2014-01-14

**Authors:** Richard Robinson

**Affiliations:** Freelance Science Writer, Sherborn, Massachusetts, United States of America

For those fascinated by cell biology, it can be delightful to imagine shrinking to the size of a bacterium to become, in Christian du Duve's word, a cytonaut, a tiny explorer inside the labyrinth of the cell. But there's a hitch: the cytoplasm of a cell is no swimming pool. It is instead crowded and viscous, with globular proteins and tangles of fibers forming a sticky gel that neither the imaginary cytonaut, nor the real bacterium, can traverse with ease.

**Figure:**
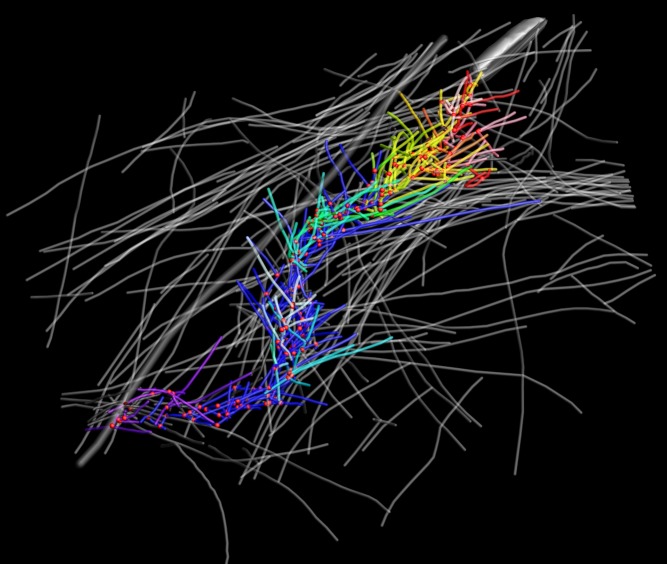
Electron tomography of baculovirus (silver cylinder in the upper right corner) reveals its propulsion mechanism through the cytoskeleton network (grey filaments) of infected cells by nucleating a fishbone-like array of branched actin filaments (colored) at the rear end.

Pathogenic bacteria and viruses of several kinds, including foodborne Listeria and Shigella, have solved the problem of getting from one side of the cell to the other by hijacking elements of the cell's own cytoskeleton. It has been clear for over two decades that these bacteria express membrane proteins that cause actin to polymerize on one side of the bacterium, forming an ever-growing scaffold of fibers (a “comet tail”) that pushes the bacterium rapidly through the sticky goo. The same mechanism can even push the bacterium out one cell and up to the edge of another, where it is engulfed, spreading the infection.

While the mechanism has been clear, details of the 3-D structure and dynamics of the actin scaffold have been less so. In this issue of *PLOS Biology*, Jan Mueller, Victor Small, and colleagues elucidate many of those details using cryo-electron tomography and a simple but powerful model of the actin-pathogen interaction.

Cryo-ET reconstructs 3-D images from multiple 2-D images taken at multiple angles, but pathogens are too large for the high-resolution images the authors were after. Instead, they used a baculovirus that also triggers comet tail formation, and which is only six times as wide as the actin fiber itself. They allowed the virus to infect a vertebrate cell, then fixed the cell, extracted the cytoskeleton, and created their images.

They found that the actin filaments of the comet tail formed a herringbone-like array, with filaments growing and diverging from a central core, and with the virus continually propelled forward by the growing fibers. Image analysis revealed that of the dozens of growing fibers near the virus, an average of four actually contacted it at any one time. Actin polymerizes from only one end, and the authors showed that it was the growing end, not the stationary end, that contacted the virus to push it along.

To better understand the constraints on this dynamic system, they built a simple 2-D mathematical model, which included the effects of cytoplasmic friction, Brownian motion, and the forces exerted by the actin filaments. They considered three alternatives to describe the actin-virus connection: either it was tethered at all times, or not at all, or only when the actin filament developed a new branch. They found that only the constantly tethered model could mimic the actual movements, and that a key stabilizing feature of the system was the pulling force exerted on the virus by a filament that is attached but not growing as fast as its neighbors. The model also showed that with too few filaments, the virus would veer rapidly off course (imagine trying to push a ball through molasses with a single stick). The straightest path required attachment of three to four filaments at any one time.

Similar herringbone patterns have been observed in comet tails from Listeria and other infectious agents, suggesting this mechanism may be a general one. These findings exclude previous models of pathogens propulsion by actin and add significant insights to understand the mechanics of pathogen movement through cells.

**Mueller J, Pfanzelter J, Winkler C, Narita A, Le Clainche V, et al. (2014) Electron Tomography and Simulation of Baculovirus Actin Comet Tails Support a Tethered Filament Model of Pathogen Propulsion.** doi:10.1371/journal.pbio.1001765

